# Prevalence and Clinical Characteristics of SARS-CoV-2 Confirmed and Negative Kawasaki Disease Patients During the Pandemic in Spain

**DOI:** 10.3389/fped.2020.617039

**Published:** 2021-01-18

**Authors:** Elisa Fernández-Cooke, Carlos D. Grasa, Sara Domínguez-Rodríguez, Ana Barrios Tascón, Judith Sánchez-Manubens, Jordi Anton, Beatriz Mercader, Enrique Villalobos, Marisol Camacho, María Luisa Navarro Gómez, Manuel Oltra Benavent, Gemma Giralt, Matilde Bustillo, Ana María Bello Naranjo, Beatriz Rocandio, Moisés Rodríguez-González, Esmeralda Núñez Cuadros, Javier Aracil Santos, David Moreno, Cristina Calvo, The KAWA-RACE Study Group

**Affiliations:** ^1^Pediatric Infectious Diseases Unit, Department of Pediatrics, Hospital Universitario 12 de Octubre, Madrid, Spain; ^2^Pediatric Research and Clinical Trials Unit (UPIC), Instituto de Investigación Sanitaria Hospital 12 de Octubre (imas12), Madrid, Spain; ^3^Pediatric Infectious Diseases Unit, Department of Pediatrics, Hospital Universitario La Paz, Madrid, Spain; ^4^Department of Pediatrics, Hospital Universitario Infanta Sofia, Madrid, Spain; ^5^Pediatric Rheumatology Department, Hospital Sant Joan de Déu, Universitat Autònoma de Barcelona, Barcelona, Spain; ^6^Department of Pediatrics, Hospital Clínico Universitario Virgen de la Arrixaca, Murcia, Spain; ^7^Department of Pediatrics, Hospital Infantil Universitario Niño Jesús, Madrid, Spain; ^8^Pediatric Infectious Diseases, Rheumatology and Immunology Unit, Department of Pediatrics, Hospital Virgen del Rocío, Sevilla, Spain; ^9^Department of Pediatrics, Hospital Universitario Gregorio Marañón, Madrid, Spain; ^10^Department of Pediatrics, Hospital Universitario y Politécnico La Fe, Valencia, Spain; ^11^Pediatric Cardiology Unit, Department of Pediatrics, Hospital Universitario Vall d'Hebron Barcelona, Barcelona, Spain; ^12^Pediatric Infectious Disease Unit, Department of Pediatrics, Hospital Universitario Miguel Servet, Zaragoza, Spain; ^13^Department of Pediatrics, Hospital Universitario Materno-Infantil de Las Palmas de Gran Canaria, Canarias, Spain; ^14^Department of Pediatrics, Hospital Universitario de Donostia, Guipuzcoa, Spain; ^15^Department of Pediatrics, Hospital Universitario Puerta Del Mar de Cádiz, Cádiz, Spain; ^16^Department of Pediatrics, Hospital Regional Universitario de Málaga, Málaga, Spain

**Keywords:** Kawasaki disease (KD), children, SARS-CoV-2, COVID-19, shock, multisystem inflammatory syndrome in children (MIS-C), pediatric inflammatory multisystem syndrome (PIMS-TS)

## Abstract

**Introduction:** COVID-19 has a less severe course in children. In April 2020, some children presented with signs of multisystem inflammation with clinical signs overlapping with Kawasaki disease (KD), most of them requiring admission to the pediatric intensive care unit (PICU). This study aimed to describe the prevalence and clinical characteristics of KD SARS-CoV-2 confirmed and negative patients during the pandemic in Spain.

**Material and Methods:** Medical data of KD patients from January 1, 2018 until May 30, 2020 was collected from the KAWA-RACE study group. We compared the KD cases diagnosed during the COVID-19 period (March 1–May 30, 2020) that were either SARS-CoV-2 confirmed (CoV+) or negative (CoV–) to those from the same period during 2018 and 2019 (PreCoV).

**Results:** One hundred and twenty-four cases were collected. There was a significant increase in cases and PICU admissions in 2020 (*P*-trend = 0.001 and 0.0004, respectively). CoV+ patients were significantly older (7.5 vs. 2.5 yr) and mainly non-Caucasian (64 vs. 29%), had incomplete KD presentation (73 vs. 32%), lower leucocyte (9.5 vs. 15.5 × 10^9^) and platelet count (174 vs. 423 × 10^9^/L), higher inflammatory markers (C-Reactive Protein 18.5vs. 10.9 mg/dl) and terminal segment of the natriuretic atrial peptide (4,766 vs. 505 pg/ml), less aneurysm development (3.8 vs. 11.1%), and more myocardial dysfunction (30.8 vs. 1.6%) than PreCoV patients. Respiratory symptoms were not increased during the COVID-19 period.

**Conclusion:** The KD CoV+ patients mostly meet pediatric inflammatory multisystem syndrome temporally associated with COVID-19/multisystem inflammatory syndrome in children criteria. Whether this is a novel entity or the same disease on different ends of the spectrum is yet to be clarified.

## Introduction

The epidemic of severe acute respiratory syndrome coronavirus 2 (SARS-CoV-2), causing COVID-19, has spread rapidly around the globe (1). Spain was one of the first European countries to be affected after Italy, with the outbreak estimated to have started in February 2020.

In contrast with adults, the disease in children appears to have a less severe course, with almost no fatalities, and those reported were mainly in children with severe underlining conditions (2, 3). But in April, some children presented critically ill with fever, shock, and signs of multisystem inflammation most of them requiring admission to the pediatric intensive care unit (PICU). They presented clinical signs overlapping with Kawasaki disease (KD) (4, 5) triggering alerts to pediatricians. Following the alert, the World Health Organization (WHO) (6), the European Centre for Disease Prevention and Control (7), the US Centers for Disease Control and Prevention (8), and the Royal Collage of Paediatrics and Child Health (9) have all produced definitions for this new entity. It was called pediatric inflammatory multisystem syndrome temporally associated with SARS-CoV-2 infection (PIMS-TS) (9) or multisystem inflammatory syndrome in children (MIS-C) (8) and case definition and guidance on clinical management was published.

The cause of KD remains unknown; however, it is suggested that an infectious agent might trigger the illness (10). A small proportion of KD patients present with Kawasaki disease shock syndrome (KDSS) resembling PIMS-TS/MIS-C (11). Cases of KD with concurrent COVID-19 infection were reported (12) suggesting that SARS-CoV-2 may trigger a cytokine storm leading to this newly defined syndrome (13–16).

This study aimed to compare the prevalence and features of KD patients before SARS-CoV-2 pandemic and compare them to the SARS-CoV-2 positive and negative cases presenting during the pandemic.

## Materials and Methods

### Network Setup

During 2015 a nationwide KD study group named KAWA-RACE was setup. Patients were included retrospectively from 2011 through 2016 and prospectively from 2018 onward, based on declaration from pediatricians of recruiting centers. A total of 93 Spanish hospitals joined the network. Our study complies with the Declaration of Helsinki and the ethics committee at Instituto de Investigación Hospital 12 de Octubre approved this study (CEIC 15/316). The inclusion of patients with KD was approved following informed consent from parents/guardians. All patient data were fully anonymized before we accessed them, and then the database was reviewed to clean inconsistencies and confirm patients' diagnoses based on information provided.

### Data Source, Collection, and Management

Prospective medical data was included from January 1, 2018 until May 30, 2020. A research electronic database capture (17) was created and sent to the participant clinicians together with the study protocol.

We established the date of March 1, 2020 as SARS-CoV-2 exposed cases, collected cases until May 30, 2020, and considered this COVID-19 period (CoV-19p). The patients' demographic, clinical, laboratory, and echocardiographic data were recorded.

### Subjects and Case Definitions

Individual patient data were reviewed to confirm the diagnosis of complete or incomplete KD according to the American Heart Association (AHA, 2017) (18). Coronary arteries measurements followed Z-score of Montreal scale (19), and the classification according to the Z-score followed AHA guidelines (18); coronary artery lesions (CAL) were considered if the Z-score > 2, and cardiac dysfunction was considered if ejection fraction was <55% (20). All patients <16 years of age diagnosed with KD were included in the study. We looked at the SARS-CoV-2-positive patients to see if they fulfilled the WHO definition of MIS-C (8) and/or the Royal College of Pediatrics and Child Health definition of PIMS-TS (9).

Two groups were established for comparison. We compared the KD cases diagnosed during the same period of 2018 and 2019 (PreCoV) to those during CoV-19p that were SARS-CoV-2 negative confirmed by both polymerase chain reaction (PCR) and serology (CoV-) and to those during CoV-19p that were SARS-CoV-2 confirmed with either PCR and/or serology (CoV+).

To assess the prevalence, we compared the same periods of 2018, 2019, and 2020 (March 1, 2020–May 30, 2020). We excluded patients older than 16 years at the time of diagnosis, those patients diagnosed from January 1 to February 29, 2020, because the virus could be circulating undetected, but tests were not routinely performed and patients in whom PCR and serology were not performed. Therefore, we could not assure they were SARS-CoV-2 negative.

### Statistical Analysis

Baseline characteristics were described through summary tables reporting frequencies and total records in case of categorical variables and median [interquartile range (IQR)] when continuous. Chi-squared and Fisher-test (low cell sizes < 5) were applied to assess differences among periods for categorical variables. For continuous variables, the non-parametric U-Mann-Whitney test was applied. Normality was tested with the Shapiro-Wilk test. The incidence was estimated using the incidence R package (21), and trend was calculated using the Chi-squared test. R software was used for all analysis (22).

## Results

**Figure 1:**
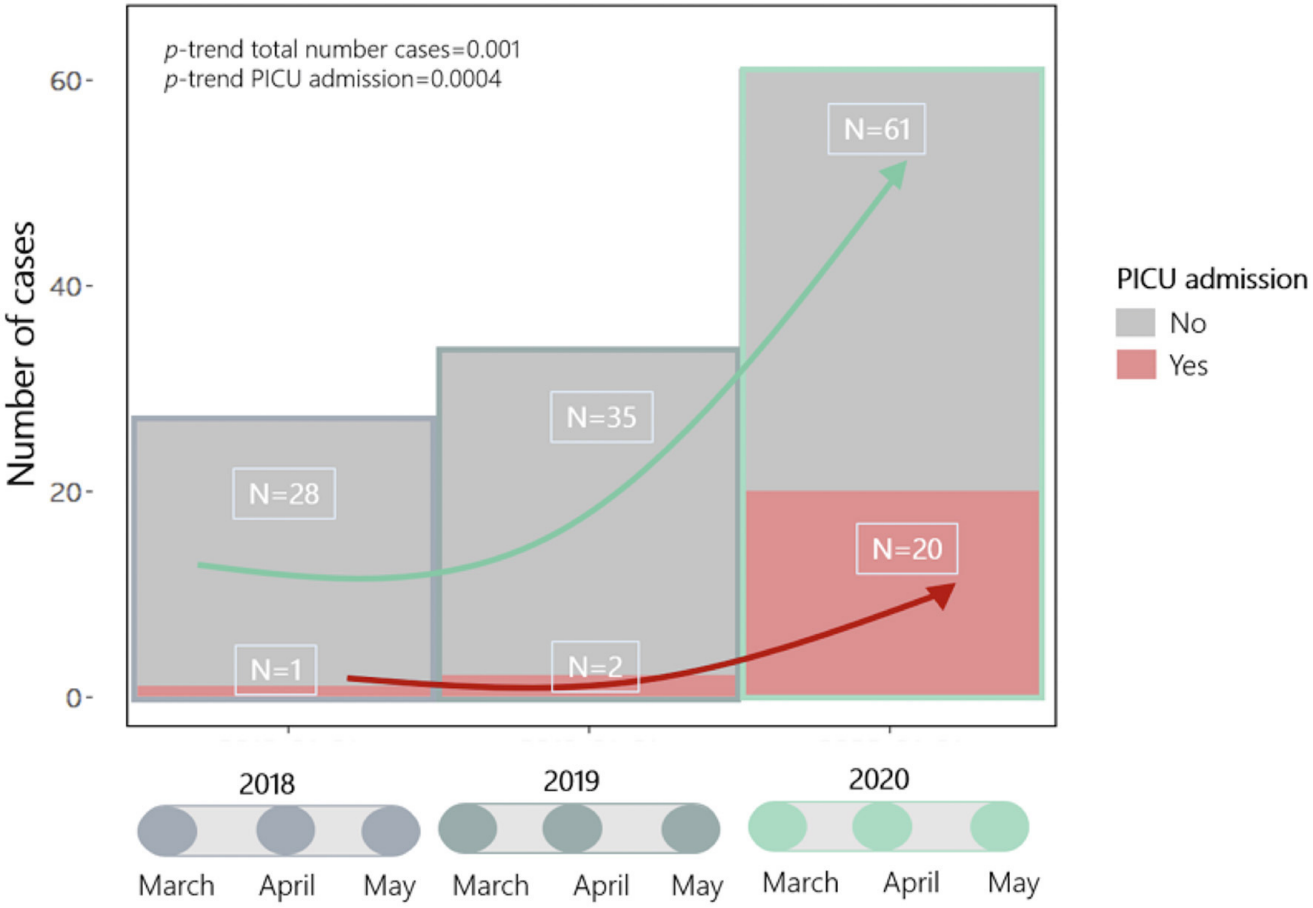
Trend in Kawasaki disease cases and PICU admissions from 1st March to 30th May during 2018–19 (preCoV-19p) and 2020 (CoV-19p).

Nationwide, 124 cases were collected during the periods March 1–May 30, 2018/19/20 with 23 (19%) requiring PICU; the diagnosis of KD was confirmed for all patients after individual data review. There was a significant increase in cases and PICU admissions in 2020 (*P*-trend = 0.001 and 0.0004, respectively) (Figure 1).

For comparison with the CoV– and CoV+ groups, we excluded 15 patients in whom PCR and serology were not performed, and therefore we could not assure they were SARS-CoV-2 negative, leaving 109 patients included for further analysis−63 in PreCoV group, 26 CoV+, and 20 CoV–. In the CoV+ group, SARS-CoV-2 PCR was positive in 42% (11/26); SARS-CoV-2 serology was positive in 100% (21/21) of patients that had the test performed, and both were positive in 29% (*n* = 6). All the patients in the CoV- group had a SARS-CoV-2 PCR and serology performed that were both negative (Tables 1, 2).

**Table 1:** Microbiological SARS-CoV-2 and blood test results of patients with Kawasaki disease (2018–2019) vs 2020 (*CoV*+*) and (CoV-)*.

	**2018–2019 *N* = 63**	**2020 (*CoV+) n* = 26**	***p*. value**	**2020 *(CoV-) N* = 20**	***p*. value^**#**^**
**Microbiological Results**					
Positive result (non-SARS-CoV-2)	17 (27%)	3 (15%)	0.227	4 (20.0%)	0.659
Coronavirus PCR performed	–	26 (100%)		20 (100%)	
Positive	–	11 (42.3%)		0 (0.00%)	
Coronavirus Serology performed	–	21 (80.8%)		20 (100%)	
Positive	–	21 (100%)^*^		0 (0.00%)	
PCR+ and Serology+	–	6 (28.6%)		0 (0.00%)	
**Blood tests**					
Hemoglobin (g/dL)	11.6 (10.6; 12.2)	12.2 (11.0; 13.3)	0.018	10.9 (10.3; 11.9)	0.547
Leucocytes × 10^9^ /L	15.5 (11.9; 20.1)	9.50 (7.40; 12.8)	<0.001	13.8 (8.98; 21.7)	0.434
Platelets at admission	423 (301; 538)	174 (118; 250)	<0.001	294 (232; 423)	0.024
Platelets (min)	371 (284; 446)	174 (118; 250)	<0.001	290 (193; 423)	0.125
Platelets (max)	605 (530; 735)	542 (394; 703)	0.064	599 (299; 711)	0.225
ESR (mm/h)	67.0 (56.5; 86.5)	46.0 (11.5; 61.0)	0.010	56.0 (33.2; 72.0)	0.113
C-reactive protein (mg/dl)	10.9 (6.81; 18.2)	18.5 (11.4; 24.2)	0.004	14.2 (9.08; 16.8)	0.450
Procalcitonin (ng/mL)	0.85 (0.39; 2.87)	4.54 (2.56; 7.63)	0.003	0.48 (0.28; 3.33)	0.640
Albumin (g/L)	3.50 (3.27; 4.00)	3.40 (3.00; 3.80)	0.153	3.65 (3.30; 3.92)	0.779
ALT (IU/L)	50.0 (16.0; 103)	24.0 (18.0; 44.0)	0.061	34.0 (20.0; 69.0)	0.662
AST (IU/L)	40.0 (27.0; 60.0)	34.0 (26.0; 56.0)	0.351	35.0 (23.0; 46.0)	0.170
GGT (IU/L)	42.0 (20.8; 114)	20.5 (15.0; 46.8)	0.031	15.0 (13.5; 66.8)	0.137
Nt proBNP	503 (356; 1,475)	4,766 (3,046; 13,596)	<0.001	776 (268; 1,260)	0.849
IL_6		185 (75.4; 310)		82.4 (51.7; 84.6)	
D-dimer	1,883 (671; 3,185)	2,461 (1,041; 3,960)	0.591	625 (287; 5,478)	0.705
Fibrinogen	597 (534; 680)	810 (518; 930)	0.289	591 (523; 619)	0.605
Ferritin	185 (128; 247)	476 (358; 966)	<0.001	153 (116; 588)	0.479

# *This p-value compares 2018–2019 with 2020 (CoV–)*.

* *Not tested in 5 patients. CoV+, SARS-CoV-2 positive patients; CoV–, SARS-CoV-2 negative patients; ESR, Erythrocyte sedimentation rate; ALT, Alanine transaminase; AST, aspartate aminotransferase; GGT, gamma-glutamyl transpeptidase; NT-proBNP, N-terminal pro b-type natriuretic peptide; IL-6, Interleukin 6*.

**Table 2:** Detailed microbiological non SARS-CoV-2 results of patients with Kawasaki disease (2018–2019), 2020 (CoV+) and (CoV-).

**2018**–**2019**		**2020**			
		**SARS-COV-2 POSITIVE**			
**Patient (*****n*** **= 63)**	**Microbiology (Non SARS-CoV-2)**	**Patient (*****n*** **= 46)**	**Microbiology (Non SARS-COV-2)**	**PCR SARS-COV-2**	**Serology SARS-COV-2**
1	(–)	1	(–)	(+)	Not tested
2	(–)	2	(–)	(–)	(+)
3	(–)	3	EBV & CMV^*d*^	(+)	Not tested
4	Enterovirus^*b*^	4	(–)	(+)	(+)
5	(–)	5	(–)	(+)	Not tested
6	(–)	6	(–)	(–)	(+)
7	Metapneumo & coronavirus^*a*^	7	(–)	(–)	(+)
8	(–)	8	(–)	(–)	(+)
9	(–)	9	(–)	(–)	(+)
10	(–)	10	(–)	(–)	(+)
11	(–)	11	(–)	(–)	(+)
12	*Salmonella*^*c*^	12	(–)	(+)	(+)
13	(–)	13	(–)	(+)	(+)
14	(–)	14	(–)	(–)	(+)
15	(–)	15	(–)	(–)	(+)
16	(–)	16	(–)	(–)	(+)
17	(–)	17	(–)	(–)	(+)
18	(–)	18	(–)	(–)	(+)
19	(–)	19	CMV & EBV in blood (+)	(+)	Not tested
20	(–)	20	(–)	(–)	(+)
21	(–)	21	Anti-HBc, & anti-HBs (+)	(+)	Not tested
22	(–)	22	(–)	(–)	(+)
23	(–)	23	(–)	(–)	(+)
24	(–)	24	(–)	(+)	(+)
25	(–)	25	(–)	(+)	(+)
26	(–)	26	(–)	(+)	(+)
27	(–)	**SARS-COV-2 NEGATIVE**			
28	(–)	27	(–)	(–)	(–)
29	(–)	28	*Mycoplasma*^*d*^	(–)	(–)
30	(–)	30	(–)	(–)	(–)
31	(–)	31	(–)	(–)	(–)
32	(–)	32	(–)	(–)	(–)
33	Rhinovirus^*a*^	33	(–)	(–)	(–)
34	(–)	34	Rhinovirus & enterovirus^*a*^^,^^*b*^	(–)	(–)
35	Parvovirus^*d*^	35	(–)	(–)	(–)
36	GAS^*b*^	36	(–)	(–)	(–)
37	Rhinovirus^*a*^	37	(–)	(–)	(–)
38	Rhinovirus^*a*^	38	Adenovirus^*a*^ & *E.coli*^*e*^	(–)	(–)
39	Rhinovirus^*a*^	39	(–)	(–)	(–)
40	(–)	40	Bocavirus^*a*^	(–)	(–)
41	(–)	41	(–)	(–)	(–)
42	(–)	42	(–)	(–)	(–)
43	(–)	43	(–)	(–)	(–)
44	(–)	44	(–)	(–)	(–)
45	(–)	45	(–)	(–)	(–)
46	GAS^*b*^	46	(–)	(–)	(–)
47	*Streptococcus* spp.^*b*^	47	(–)	(–)	(–)
48	(–)				
49	(–)				
50	GAS^*b*^				
51	(–)				
52	Parvovirus^*d*^				
53	(–)				
54	*Mycoplasma*^*d*^				
55	(–)				
56	(–)				
57	(–)				
58	(–)				
59	(–)				
60	(–)				
61	CMV^*d*^				
62	Adenovirus^*a*^				
63	CMV^*d*^				

a *Nasopharyngeal aspirate*.

b *Pharyngeal swab*.

c *Stool culture*.

d *Serology (IgM)*.

e *Urine culture*.

*(–), Negative; (+), Positive; GAS, Group A Streptococcus; CMV, Cytomegalovirus; EBV, Epstein Barr Virus; Anti-HBc, hepatitis B core antibody; Anti-HBs, hepatitis B surface antibody*.

*All patients from the CoV– group were tested for SARS-COV-2 PCR and serology*.

### Clinical Characteristics (Table 3)

The median PreCoV age was 2.55 (IQR, 1.5–3.9), 3.56 (IQR, 2.2–6.4) for CoV– and significantly higher 7.54 (IQR, 5.4–10.8) for CoV+ patients (*p* < 001). There was a male predominance PreCoV (62%) and in CoV+ (69%) with a 1:1 ratio in CoV- patients. We found that in PreCoV and CoV– patients, around one-fourth of them were non-Caucasian (18/63, 29%, and 5/20, 25%, respectively), while in CoV+ patients this was significantly higher (16/26, 64%). The median duration of fever was 7 days in all groups.

**Table 3:** Demographics and Clinical Features of patients with Kawasaki disease (2018–2019) vs. 2020 (*CoV*+*) and (CoV*–).

	**2018–2019 *N =* 63**	**2020 (*CoV+) n =* 26**	***p*. value**	**2020 *(CoV-) N = 20***	***p*. value**
**Demographics**					
Male	38 (62.3%)	18 (69.2%)	0.709	10 (50.0%)	0.478
Median age (years) (IQR)	2.55 (1.46; 3.89)	7.54 (5.36; 10.8)	<0.001	3.56 (2.20; 6.37)	0.074
Non-Caucasian	18 (28.6%)	16 (64.0%)	0.005	5 (25.0%)	0.981
**Clinical features**					
Median days of fever	7.00 (6.00; 8.00)	7.00 (6.00; 9.00)	0.483	7.00 (5.75; 8.00)	0.817
Complete KD	43 (68.3%)	7 (26.9%)	<0.001	8 (40.0%)	0.019
***Classical criteria***					
Conjunctival injection	49 (77.8%)	22 (84.6%)	0.660	16 (80.0%)	1.000
Lips and oral changes	53 (85.5%)	13 (50.0%)	0.001	16 (80.0%)	0.725
Changes in extremities	41 (66.1%)	8 (32.0%)	0.008	7 (35.0%)	0.028
Polymorphous exanthema	54 (87.1%)	21 (80.8%)	0.515	14 (70.0%)	0.094
Acute lymphadenopathy	32 (50.8%)	11 (42.3%)	0.620	7 (35.0%)	0.329
***Other symptoms***					
**Respiratory symptoms**	40 (63.5%)	19 (73.1%)	0.533	13 (65.0%)	1.000
Rhinorrhea	21 (33.3%)	0 (0.00%)	0.002	2 (10.0%)	0.081
Cough	15 (23.8%)	4 (15.4%)	0.550	3 (15.0%)	0.540
Wheezing	2 (3.17%)	1 (3.85%)	1.000	1 (5.00%)	0.568
Pleural effusion	2 (3.17%)	1 (3.85%)	1.000	0 (0.00%)	1.000
Dyspnea	2 (3.17%)	1 (3.85%)	1.000	0 (0.00%)	1.000
Abnormal Chest-X-ray	6 (17.6%)	10 (38.5%)	0.131	4 (25.0%)	0.707
**Musculoskeletal symptoms**					
Arthralgia	8 (12.7%)	1 (3.85%)	0.274	1 (5.00%)	0.680
Arthritis	8 (12.7%)	1 (3.85%)	0.274	1 (5.00%)	0.680
Myalgia	4 (6.35%)	3 (11.5%)	0.412	0 (0.00%)	0.568
Gastrointestinal symptoms	36 (57.1%)	17 (65.4%)	0.629	11 (55.0%)	1.000
Abdominal pain	21 (33.3%)	15 (57.7%)	0.059	6 (30.0%)	0.997
Nausea	4 (6.35%)	3 (11.5%)	0.412	2 (10.0%)	0.628
Vomits	23 (36.5%)	10 (38.5%)	1.000	5 (25.0%)	0.498
Diarrhea	8 (12.7%)	6 (23.1%)	0.336	4 (20.0%)	0.471
Any hepatic alteration	30 (47.6%)	14 (53.8%)	0.763	9 (45.0%)	1.000
Hypertransaminasemia	30 (47.6%)	13 (50.0%)	1.000	8 (40.0%)	0.735
Hyperbilirubinemia	4 (6.35%)	1 (3.85%)	1.000	0 (0.00%)	0.568
Hepatomegaly	4 (6.35%)	2 (7.69%)	1.000	0 (0.00%)	0.568
Cholestasis	1 (1.59%)	1 (3.85%)	0.501	0 (0.00%)	1.000
**Nervous system symptoms**					
Headache	6 (9.68%)	3 (11.5%)	0.688	1 (5.26%)	1.000
Irritability	35 (55.6%)	1 (3.85%)	<0.001	9 (47.4%)	0.271
Aseptic meningitis	2 (3.17%)	1 (3.85%)	0.328	0 (0.00%)	0.707
**Genitourinary symptoms**					
Hematuria	4 (6.35%)	2 (7.69%)	0.680	3 (15.0%)	0.218
Sterile pyuria	13 (21.3%)	0 (0.00%)	0.015	7 (35.0%)	0.427
**Shock not related with IVIG**	1 (1.59%)	13 (50.0%)	<0.001	4 (20.0%)	0.011
**PIMS-TS criteria fulfilled**	–	23 (88.5%)		8 (40%)	
**Complete KD**		7/23 (30.4%)		3/8 (37.5%)	
**Incomplete KD**		16/23 (69.6%)		5/8 (62.5%)	
**MIS-C criteria fulfilled**	–	23 (88.5%)		9 (45%)	
**Complete KD**		7/23 (%)		2/9 (22.2%)	
**Incomplete KD**		16/23 (%)		7/9 (77.8%)	
**Cardiology examinations**					
Any echocardiogram alterations	23 (37.1%)	13 (50.0%)	0.376	9 (45%)	0.594
Coronary artery lesions	18 (75.0%)	4 (30.8%)	0.023	3 (33.3%)	0.044
Ectasia	12 (19.0%)	3 (11.5%)	0.538	2 (10.0%)	0.500
Aneurysm	7/23 (30.4%)	1/13 (7.7%)^*^	0.212	2/9 (22.2%)	1.000
	7/63 (11.1%)	1/26 (3.85%)	0.429	2/20 (10.0%)	1.000
z score: (small)	7 (100%)	1 (100%)		2 (100%)	.
Persistent Aneurysm	1/7 (14.3%)	0 (0.00%)	0.250	0 (0.00%)	0.417
Systolic dysfunction Left V.	1/63 (1.59%)	8/26 (30.8%)	<0.001	2/20 (10.0%)	0.143
Pericardial effusion	13/23 (56.5%)	3/13 (23.1%)	0.083	4/9 (44.4%)	0.699
Repolarization alterations	2 (3.17%)	5 (19.2%)	0.021	0 (0.00%)	1.000
**Pharmacotherapy**					
Days from fever to IVIG	6.00 (5.00; 8.00)	6.00 (4.00; 7.00)	0.194	6.00 (5.00; 8.25)	0.455
IVIG 1st dose	58/63 (92%)	21/26 (80.8%)	0.149	20 (100%)	0.329
IVIG 2nd dose	8/58 (13.8%)	4/21 (19.0%)	0.723	4 (20.0%)	0.492
Corticosteroids	18/63 (28.6%)	16/26 (61.5%)	0.008	10 (50.0%)	0.104
Tocilizumab	0 (0.00%)	1 (33.3%)	1.000	1 (100%)	1.000
Anakinra	0 (0.00%)	1 (14.3%)	1.000	0	–
**Outcome**					
Admission to PICU	3 (4.92%)	13 (50.0%)	<0.001	6 (30.0%)	0.006
Days in PICU	7.00 (5.50; 7.50)	4.00 (3.00; 5.00)	0.247	4.50 (2.25; 6.75)	0.362
**Reason PICU admission**					
Myocarditis	0 (0.00%)	6 (23.1%)	<0.001	1 (5.00%)	0.241
Cardiac dysfunction	0 (0.00%)	6 (23.1%)	<0.001	0	–
Suspicion of Sepsis	2 (3.17%)	1 (3.85%)	1.000	0 (0.00%)	1.000
Hypotension shock	1 (1.59%)	9 (34.6%)	<0.001	3 (15.0%)	0.042
Vasoactive drugs:	0 (0.00%)	3 (11.5%)	0.023	1 (5.00%)	0.241
Cardiogenic shock	0 (0.00%)	1 (3.85%)	0.292	0	-
Respiratory distress	1 (1.59%)	0 (0.00%)	1.000	0 (0.00%)	1.000

*Median (interquartilic range). CoV+, SARS-CoV-2 positive patients; CoV–, SARS-CoV-2 negative patients; IQR, interquartile range; KD, Kawasaki Disease; IVIG, intravenous immunoglobulin; Left V, Left Ventricle; MIS-C, multisystem inflammatory syndrome in children; PICU, pediatric intensive care unit; PIMS-TS, pediatric inflammatory multisystemic syndrome temporary associated with SARS-CoV-2*.

* *This patient received IVIG within the first 10 days after fever onset*.

Complete KD was less frequent during the CoV-19p [(CoV– 8/20, 40%) and (CoV+ 7/26, 27%) vs. PreCoV (43/63, 68%)]. Of note during the CoV-19p, especially in the CoV+ group, up to 23% of patients were suspected and treated as KD but did not fulfill AHA criteria (complete or incomplete), while during the PreCoV this did not happen. Patients from CoV-19p who did not fulfill AHA criteria when treatment was administered, finally fulfilled criteria for incomplete KD.

Patients CoV+ presented with significantly fewer lips and oral changes than PreCoV patients (85 vs. 50%, *p* < 0.005). No patient in the CoV+ group had sterile pyuria while it was observed in 21% of PreCoV and 35% of CoV– patients. Respiratory symptoms were not increased during COV-19p, and although it was more likely to have an abnormal chest X-ray in the CoV+ group, this was not significant. Overall, gastrointestinal symptoms were observed in 57% PreCoV vs. 65% in CoV+ and 55% CoV– with an increased proportion of patients with abdominal pain in the CoV+ group (33 vs. 58% and 30%). Irritability was significantly lower in the CoV+ group. Shock that was not related to IVIG infusion was observed in an increased number of patients during the CoV-19p (CoV– 4/20, 20%, and CoV+ 13/26,50%, *P* < 0.001) vs. 1.6% of patients during the PreCoV. Twenty-three (88.5%) of the CoV+ patients fulfilled both PIMS-TC and MIS-C criteria; from the CoV– cohort, 45% of patients fulfilled the criteria for MIS-C, and 40% for PIMS-TS, assuming all these patients would have had exposure to SARS-CoV-2 during this period, which is not recorded in the database (Tables 4A, B).

**TABLE 4A:** Patients from CoV+ cohort, indicating complete/incomplete KD and the criteria met for PIMS-TS and MIS-C diagnosis.

**Patient number**	**1**	**2**	**3**	**4**	**5**	**6**	**7**	**8**	**9**	**10**	**11**	**12**	**13**	**14**	**15**	**16**	**17**	**18**	**19**	**20**	**21**	**22**	**23**	**24**	**25**	**26**
Complete/Incomplete KD	C	C	I	I	I	C	C	^*^	^*^	C	I	C	I	I	^*^	I	I	I	^*^	I	I	I	C	I	I	^*^
Fever > 3 d	x	x	x	x	x	x	x	x	x	x	x	x	x	x	x	x	x	x	x	x	x	x	x	x	x	x
Rash/Conjunctivitis/Mucocutaneous inflammation signs	x	x	x	x	x	x	x	x	x	x		x	x	x	x	x	x	x	x	x		x	x	x	x	
Hypotension/Shock	x	x	x			x	x							x	x	x				x	x	x	x			x
Myocardial dysfunction/pericarditis/valvulitis/CAA	x	x	x			x	x	x	x	x	x	x		x	x	x		x	x	x	x	x		x	x	x
Coagulopathy		x	x	x		x	x			x				x	x	x	x		x		x	x	x			
GI symptoms		x	x	x	#	x	x	#				‡		x	x	x	x	x	x	x	x	x		x	x	x
PCR > 5 mg/dL		x	x	x		x	x	x	x	x	x	x	x	x	x	x	x	x	x	x	x	x	x	x	x	x
PCT > 1 ng/mL	x	x	x	x			x	x	x	x		x	x	x	x	x		x	x	x	x				x	x
No other cause	x	x	x	x	x	x	x	x	x	x	x	x	x	x	x	x	x	x	x	x	x	x	x	x	x	x
**PIMS-TS (WHO)**	Yes	Yes	Yes	Yes	No	Yes	Yes	Yes	Yes	Yes	No	Yes	No	Yes	Yes	Yes	Yes	Yes	Yes	Yes	Yes	Yes	Yes	Yes	Yes	Yes
Persistent fever	x	x	x	x	x	x	x	x	x	x	x	x	x	x	x	x	x	x	x	x	x	x	x	x	x	x
Persistent inflammation	x	x	x	x	x	x	x	x	x	x	x	x	x	x	x	x	x	x	x	x	x	x	x	x	x	x
Single or multi-organ dysfunction	x	x	x	x	#	x	x	#‡	x	x	x	x		x	x	x	x	x	x	x	x	x	x	x	x	x
Shock	x	x	x			x	x							x	x	x				x	x	x	x			x
Cardiac disorder		x	x			x	x		x		x			x	x	x		x	x	x	x	x		x	x	
Respiratory disorder																			x	x						x
Renal disorder																										
GI disorder		x	x	x	#	x	x	#‡				‡		x		x	x	x	x	x	x	x		x	x	x
Neurological disorder										x	x	x														
No other cause	x	x	x	x	x	x	x	x	x	x	x	x	x	x	x	x	x	x	x	x	x	x	x	x	x	x
SARS-CoV-2	+	+	+	+	+	+	+	+	+	+	+	+	+	+	+	+	+	+	+	+	+	+	+	+	+	+
**MIS-C (RCPCH)**	Yes	Yes	Yes	Yes	No	Yes	Yes	No	Yes	Yes	Yes	Yes	No	Yes	Yes	Yes	Yes	Yes	Yes	Yes	Yes	Yes	Yes	Yes	Yes	Yes

*BP, Blood pressure; C, Complete; CAA, Coronary artery abnormalities; GI, Gastrointestinal; I, Incomplete; KD, Kawasaki disease; MIS-C, Multisystem inflammatory syndrome in children; PIMS-TS, Pediatric inflammatory multisystem syndrome temporally associated with SARS-CoV-2; RCPCH, Royal College of Paediatrics and Child Health; WHO, World Health Organization*.

* *These patients didn't meet the criteria for KD at the moment of admission, but they fulfilled the criteria for incomplete KD during admission*.

# *These patients complained only of mild abdominal pain*.

‡ *These patients had elevation of liver enzymes but <2 upper limit of normality*.

**TABLE 4B:** Patients from CoV- cohort indicating complete/incomplete KD and the criteria met for PIMS-TS and MIS-C diagnosis.

**Patient number**	**1**	**2**	**3**	**4**	**5**	**6**	**7**	**8**	**9**	**10**	**11**	**12**	**13**	**14**	**15**	**16**	**17**	**18**	**19**	**20**
Complete/Incomplete KD	I	I	C	C	I	I	C	*	I	I	I	I	C	I	C	I	C	I	C	C
Fever > 3 d	x	x	x	x	x	x	x	x	x	x	x	x	x	x	x	x	x	x	x	x
Rash/ Conjunctivitis/Mucocutaneous inflammation signs	x	x	x	x	x	x	x	x	x	x		x	x	x	x	x	x	x	x	x
Hypotension/Shock		x						x	x									x		
Myocardial dysfunction/pericarditis/valvulitis/ CAA	x	x		x	x			x			x		x		x	x	x	x		
Coagulopathy		x												x	x			x		
GI symptoms	x	x			x	x		x					x		x	x	x	x	x	
PCR> 5 mg/dL	x	x		x		x		x	x	x	x	x	x	x	x	x	x	x	x	x
PCT > 1 ng/mL								x	x						x			x		x
No other cause	x	x	x	x	x	x	x	x	x	x	x	x	x	x	x	x	x	x	x	x
**MIS-C (WHO)**	Yes	Yes	No	No	No	No	No	Yes	No	No	No	No	Yes	No	Yes	Yes	Yes	Yes	No	No
Persistent fever	x	x	x	x	x	x	x	x	x	x	x	x	x	x	x	x	x	x	x	x
Persistent inflammation	x	x	x	x	x	x	x	x	x	x	x	x	x	x	x	x	x	x	x	x
Single or multi-organ dysfunction	x	x			x			x	x		x		x		x	x		x		‡
Shock		x						x	x									x		
Cardiac disorder	x	x						x			x		x		x	x		x		
Respiratory disorder													x		x					
Renal disorder																				
GI disorder	x	x			x	#		#					x		x	x		x		‡
Neurological disorder																				
No other cause	x	x	x	x	x	x		x	x	x	x	x	x	x	x	x	x	x	x	x
SARS-CoV-2	-	-	-	-	-	-	-	-	-	-	-	-	-	-	-	-	-	-	-	-
**PIMS-TS (RCPCH)**	Yes	Yes	No	No	Yes	No	No	No	Yes	No	Yes	No	Yes	No	Yes	Yes	No	Yes	No	No

*BP, Blood pressure; C, Complete; CAA, Coronary artery abnormalities; GI, Gastrointestinal; I, Incomplete; KD, Kawasaki disease; MIS-C, Multisystem inflammatory syndrome in children; PIMS-TS, Pediatric inflammatory multisystem syndrome temporally associated with SARS-CoV-2; RCPCH, Royal College of Paediatrics and Child Health; WHO, World Health Organization*.

* *These patients didn't meet the criteria for KD at the moment of admission, but they fulfilled the criteria for incomplete KD during admission*.

# *These patients complained only of abdominal pain*.

‡ *These patients had elevation of liver enzymes but <2 upper limit of normality*.

**Diagnostic criteria of inflammatory:**

•MIS-C (WHO):

° Children and adolescents 0–19 years of age with fever > 3 days AND two of the following:

*a) Rash or bilateral non-purulent conjunctivitis or mucocutaneous inflammation signs (oral, hands, or feet)*.

*b) Hypotension or shock*.

c) Features of myocardial dysfunction, pericarditis, valvulitis, or coronary abnormalities (including ECHO findings or elevated troponin/NT-proBNP)

*d) Evidence of coagulopathy (by PT, PTT, elevated D-Dimers)*.

e) Acute gastrointestinal problems (diarrhea, vomiting, or abdominal pain).

*° AND elevated markers of inflammation such as ESR, C-reactive protein, or procalcitonin*.

*° AND no other obvious microbial cause of inflammation, including bacterial sepsis, staphylococcal or streptococcal shock síndrome*.

° AND evidence of COVID-19 (RT-PCR, antigen test, or serology positive), or likely contact with patients with COVID-19.

• PIMS-TS (RCPCH):

*° A child presenting with persistent fever, inflammation (neutrophilia, elevated CRP and lymphopaenia) and evidence of single or multi-organ dysfunction (shock, cardiac, respiratory, renal, gastrointestinal or neurological disorder) with other additional clinical, laboratory or imagining and ECG features. Children fulfilling full or partial criteria for Kawasaki disease may be included*.

*° Exclusion of any other microbial cause, including bacterial sepsis, staphylococcal or streptococcal shock syndromes, infections associated with myocarditis such as enterovirus*.

*° SARS-CoV-2 PCR testing positive or negative*.

**Figure 2:**
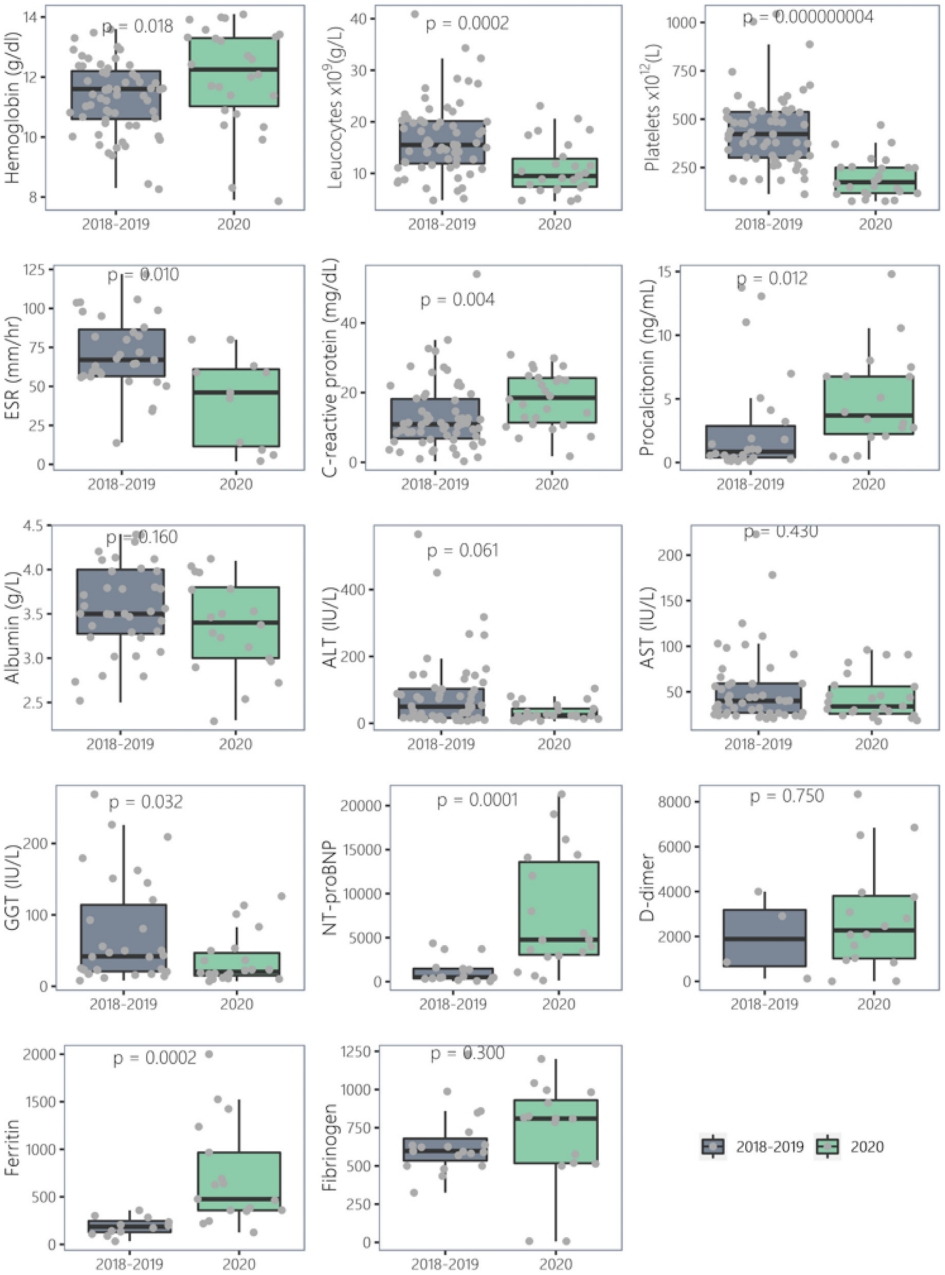
Comparison of laboratory results of Kawasaki disease cases (2018–2019) vs. 2020 (CoV+).

**Figure 3:**
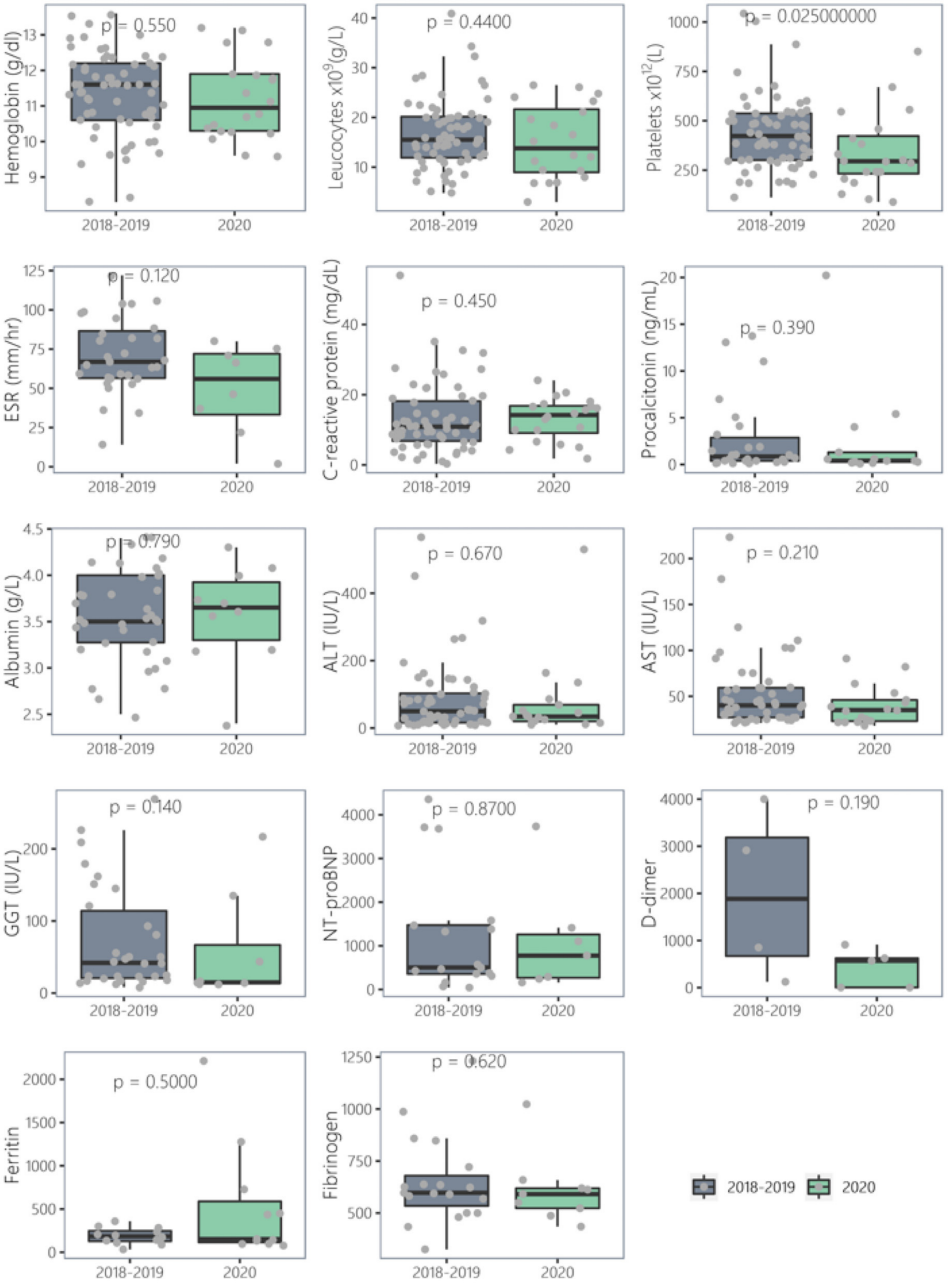
Comparison of laboratory results of Kawasaki disease cases (2018–2019) vs. 2020 (CoV–).

There were no meaningful differences in blood results between PreCoV and CoV– patients. Nevertheless, when comparing PreCoV to CoV+ patients we found in CoV+ group significantly lower leucocyte (15.5 × 10^9^/L vs. 9.5 × 10^9^/L, *p* < 0.001) and platelet count (423 × 10^9^/L vs. 174 × 10^9^/L, *p* < 0.001) and higher terminal segment of the natriuretic atrial peptide (NT-proBNP) (503 pg/ml vs. 4,766 pg/ml, *p* < 0.001), ferritin (185 ng/ml and 476 ng/ml, *p* < 0.001), C-reactive protein (CRP) (median, 10.9 vs. 18.5 mg/dl, *p* < 0.005), and procalcitonin (PCT) (median, 0.85 vs. 4.54 ng/ml, *p* < 0.005) (Table 1, Figures 2, 3).

A positive microbiological finding other than SARS-CoV-2 was found in PreCoV, CoV+, and CoV– patients in 27, 15, and 20% of cases, respectively (Table 2).

### Outcome and Treatment

Echocardiographic examinations were abnormal in PreCoV, CoV–, and CoV+ patients in 37% (23/67), 45% (9/20), and 50% (13/26), respectively: CAL were observed in 18/23 (75%), 3/9 (33.3%), and 4/13 (39%) among those with abnormal echocardiography, coronary aneurysms in 7/63 (11%), 2/20 (10%), and 1/26 (3.8%), left ventricular dysfunction in 1/63 (1.59%), 2/20 (10%), and 8/26 (30.8%), and pericardial effusion in 13/23 (56.5%), 4/9 (44.4%), and 3/13 (23.1%). Left ventricular dysfunction was the only echocardiographic finding that was significantly higher in CoV+ than PreCoV group (*p* < 0.001) and in all cases it was transient. No giant coronary artery aneurysms were seen. A higher rate of abnormal repolarization in electrocardiographic studies was observed in CoV+ patients compared with PreCoV and CoV– patients (19 vs. 3% and 0%, respectively).

Treatment with IVIG was given to 95, 100, and 80% of the PreCoV, CoV–, and CoV+ patients, respectively. The mean days of fever onset to IVIG administration were 6 days in all groups. A second IVIG dose was given in 14% (8/58), 20% (4/20), and 19% (4/21), and corticosteroids were given in 28.6% (18/63), 50% (10/20), and 61.5% (16/26), respectively. Only one CoV+ and one CoV– patient received Tocilizumab, and one patient in the CoV+ group received Anakinra.

A significantly higher number of patients were admitted to PICU from the CoV+ 50% (13/26) vs. the PreCoV group 5% (3/63), *p* < 0.001. When comparing the PreCoV to the CoV– patients, there is still a higher non-significant number of patients that require PICU 5% (3/63) vs. 30% (6/20). The main reason for PICU admission in the CoV+ group was due to cardiac dysfunction or shock. Vasoactive support was given to 3/13 (23%) in the CoV+ group vs. 1/6 (16%) in the CoV– group and none during the PreCoVp.

Only one patient from PreCoV had a persistent aneurysm, and one in the CoV– is still under follow-up. No deaths were reported (Table 3).

## Discussion

To our knowledge, this is the first study to compare KD cases prospectively recruited presenting before and during the pandemic and that study separately the SARS-CoV-2 confirmed positive and negative cases and compare them with pre-pandemic patients from the preceding years.

We report a significant increase in the number of KD cases in Spain during the SARS-CoV-2 pandemic, with an overall 2-fold increase in cases reported as KD in the national database and a 10–20-fold increase of cases admitted to PICU compared to the previous 2 years. A similar study from northern Italy found a 30-fold increased incidence (13). Despite a 2-fold increase in the cases reported as KD in the national database when we analyzed each case in this new context, we find that classical KD remains similar, as the prevalence of CoV–KD seems not to have increased. Moreover, our series provides data strongly suggesting that even in the era of COVID-19 some classical KD remain and must not be considered as PIMS.

The majority of the KD SARS-CoV-2 confirmed cases fulfilled the PIMS-TC/MIS-C criteria; knowing case definition was intended to be sensitive, and therefore most KD cases are included. Albeit a positive result, detecting SARS-CoV-2 is suggestive for PIMS-TS/MIS-C, but it is not essential for diagnosis: a likely contact with patients with COVID-19 is enough for PIMS-TS, and MIS-C criteria include negative PCR for SARS-CoV-2.

Clinical and laboratory features of CoV+ KD patients resemble PIMS-TC/MIS-C and have many significant differences from PreCoV cases. Patients with CoV+ KD are older, have non-Caucasian predominance, more intense inflammation, and greater myocardial injury than patients with classical KD.

We have compared our data to other European KD series that also looked at KD and Kawasaki-like cases presenting during the pandemic. Table 5 summarizes data from Pouletty et al. (15), Verdoni et al. (13), and Toubiana et al. (23).

**TABLE 5:** Summary from Kawasaki disease series during the SARS-CoV-2 pandemic.

	Fernández-Cooke et al.^*^ Our Cohort COVID + *N* = 26	Fernández-Cooke et al.^*^ Our Cohort COVID – *N* = 20	Pouletty et al. (15)^*^N = 16	Verdoni et al. (13)^**^N = 10	Toubiana et al. (23)^***^N = 21
Median age (years)	7.54 (5.36–10.8)	3.56 (2.2–6.37)	10 (4.7–12.5)	7.5 (3.5)	7.9 (3.7–16.6)
Gender-male *n* (%)	18 (69.2%)	10 (50%)	8 (50%)	7 (70%)	9 (43%)
**Ethnicity**					
- Caucasian	9 (36%)	15 (75%)	4 (25%)^‡^	–	12 (29%)^‡‡^ (*n* = 42)
- Not Caucasian	16 (64%)	5 (25%)	–	–	–
**Symptoms**					
Fever	26 (100%)	20 (100%)	16 (100%)	10 (100%)	21 (100%)
Polymorphous exanthema	21 (80.8%)	14 (70%)	13 (81%)	8 (80%)	16 (76%)
Conjunctival injection	22 (84.6%)	16 (80%)	15 (94%)	9 (90%)	17 (81%)
Lips and oral changes	13 (50%)	16 (80%)	14 (87%)	7 (70%)	16 (76%)
Changes in extremities	8 (32%)	7 (35%)	11 (68%)	5 (50%)	10 (48%)
Cervical lymphadenopathy	11 (42.3%)	7 (35%)	6 (37%)	1 (10%)	12 (57%)
Complete KD	7 (26.9%)	8 (40%)	10 (62%)	5 (50%)	11 (52%)
Gastrointestinal symptoms	17 (65.4%)	11 (55.0%)	13 (81%)	6 (60%)	21 (100%)
Respiratory symptoms	19 (73.1%)	13 (65.0%)	2 (12%)	–	–
Neurological symptoms	–	–	9 (56%)	–	12 (57%)
Musculoskeletal symptoms	–	–	1 (6%)	4 (40%)	2 (10%)
**Imaging**					
Abnormal chest X-ray	10 (38.5%)	4 (25%)	5 (31%)	5 (50%)	8 (44%) (*n* = 18)
**Blood tests**					
Hemoglobin (g/dL)	12.2 (11–13.3)	10.9 (10.3–11.9)	–	11 (1.2)	8.6 (5.3–12.2)
Leucocytes (×10^9^/L)	9.5 (7.4–12.8)	13.8 (8.98–21.7)	11.5 (9–14.4)	10.8 (6.1)	17.4 (5.4–42.8)
Platelets (×10^9^/L)^‡‡‡^	174 (118–250)	290 (193–423)	188 (164–244)	130 (32)	499 (78–838)
C-Reactive Protein (mg/dL)	18.5 (11.4–24.2)	14.2 (9.08–16.8)	20.7 (16.2–23.6)	25 (15.3)	25.3 (8.9–36.3)
Procalcitonin (ng/mL)	4.54 (2.56–7.63)	0.48 (0.28–3.33)	–	–	22.5 (0.1–448)
ESR (mm/h)	46 (11.5–61)	56 (33.2–72)	–	72 (24) (n=8)	–
Albumin (g/dL)	3.4 (3–3.8)	3.65 (3.3–3.92)	2.1 (1.9–2.3)	3.2 (0.3)	2.1 (1.6–3.7)
ALT (IU/L)	24 (18–44)	34 (2–69)	–	119 (217)	70 (6–257)
AST (IU/L)	34 (26–56)	35 (23–46)	–	87 (70)	–
GGT (IU/L)	20.5 (15–46.8)	15 (13.5–66.8)	–	–	59 (10–205)
NT-proBNP (ng/L)	4,766 (3,046–13,596)	776 (268–1,260)	BNP ng/L (4,319, 2,747–6,493)	1,255 (929)	BNP ng/L (3,354, 16–16,017) (*n* = 18)
D-dimer (mcg/L)	2,461 (1,041–3,960)	625 (287–5,478)	–	3,798 (1,318)	4,025 (350–19,330) (*n* = 20)
Interleukin 6 (pg/mL)	185 (75.4–310)	82.4 (51.7–84.6)	270 (136–526)	177.1 (137.4) (*n* = 4)	170 (4–1,366) (*n* = 17)
Fibrinogen (mg/dL)	810 (518–930)	591 (523–619)	–	621 (182)	–
Ferritin (ng/mL)	476 (358–966)	153 (116–588)	1,067 (272–1,709)	1,176 (1,032)	–
**Microbiological results**					
SARS-CoV-2 PCR +	11 (42.3%)	0	9 (56)	2 (20%)	8 (38%)
SARS-CoV-2 serology (IgG and/or IgM) +	21 (100%) (*n* = 21)	0	7/8 (87%)	8 (80%)	19 (90%) (*n* = 20)
**Pharmacotherapy**					
IVIG n (%)	21 (80.8%)	20 (100%)	15 (93%)	10 (100%)	21 (100%)
Steroids	16 (61.5%)	10 (50%)	4 (25%)	8 (80%)	10 (48%)
Resistance to IVIG (persistence of fever >36 h)	4 (19%) (*n* = 21)	4 (20%)	10 (62%)	10 (100%)	5 (24%)
**Cardiology examinations**					
Abnormal echocardiography	13 (50%)	9 (47.4%)	11 (69%)	6 (60%)	–
Coronary artery dilations	3 (11.5%)	2 (10%)	3 (19%)	–	5 (24%)
Coronary artery aneurisms	1 (3.85%)	2 (10%)	0	2 (20%)	0
Myocarditis	6 (23.1%)	1 (5%)	7 (43.8%)	5 (50%)^§^	16 (76%)
Pericarditis / pericardial effusion	3 (23.1%)	4 (44.4%)	4 (25%)	4 (40%)	10 (48%)
**Outcome**					
Admission to PICU	13 (50%)	6 (30%)	7 (44%)	–	17 (81%)
Shock	13 (50%)	4 (20%)	7 (44%)	5 (50%)	12 (57%)

* *Median (interquartilic range)*.

** *Results as mean (standard deviation)*.

*** *Results as median (range)*.

† *From Europe, not indicated Caucasian*.

‡‡ *Parents, not patients, and from Europe, not indicated Caucasian*.

‡‡‡ Corresponding in the different studies to:

*- Cooke et al. COVID+ Platelets at admission are equal to minimum platelets*.

*- Cooke et al. COVID- Minimum platelets (at admission 294, 193–423)*.

*- Pouletty et al. Minimum platelets*.

*- Verdoni et al. not indicated*.

*- Toubiana et al. not indicated*.

§ *Indicated ejection fraction <55%*.

*ALT, Alanine Aminotransferase; AST, Aspatate Aminotransferase; ESR, Erythrosedimentation Rate; GGT, Gamma Glutamyltransferase; IVIG, intravenous immunoglobulin; KD, Kawasaki Disease; NT-proBNP, N-Terminal proB-type Natriuretic Peptide; PICU, Pediatric Intensive Care Unit*.

The median PreCoV age was 2.55 years that was similar to the CoV– group (3.56 yr) and to our retrospective data (24) and significantly higher for CoV+ patients (7.54 yr) that was closer in age to those reported in the PIMS-TC series (9 yr) (5, 25) and the KD series during the pandemic (7.5–10 yr) (13, 15, 26–28).

Historically, KD has known to have male predominance (18), and we found a male predominance in PreCoV and in CoV+ patients. African Americans have been affected by the COVID-19 pandemic at a disproportionately higher rate (29).

Interestingly, we found a significantly higher proportion of non-Caucasians in the CoV+ group, which does not represent the general Spanish population. Additionally, our retrospective study on the same population (2011–2016) found 76% of KD cases had European origin (24). Other studies have also found over-representation of non-Caucasian patients in the KD series during the pandemic (5, 15, 23) reaching in a published PIMS-TC cohort 100% of patients (25), and this may suggest an effect of either social and living conditions or genetic susceptibility (30).

The CoV+ group had more likely incomplete KD than in the PIMS-TS series (5, 23, 31). This phenomenon could be because PIMS-TC and CoV+ KD are a separate entity or because pediatricians are more aware now and diagnose more incomplete KD in this context. After conjunctival injection, the most frequent classical symptom was erythematous rash and significantly less common lips and oral changes in CoV+ patients; this was also observed in PIMS-TC patients (5, 23, 31). No patient in the CoV+ group had sterile pyuria that is typically observed in classical KD patients.

Respiratory symptoms were not increased in our series, and although not significant, it was more likely to have an abnormal chest X-ray during the pandemic as reported by Toubiana et al. (23) (44%). Probably the small sample size in our series does not allow us to detect significant differences. There was an increased proportion of patients with abdominal pain in the CoV+ group, and this symptom was widely observed in PIMS-TC patients (5, 25). Irritability was significantly lower in the CoV+ group, probably due to the higher median age in this group. Shock that was not related to IVIG infusion was observed in a significantly increased number of patients who were CoV+. This phenomenon was also observed by the other KD pandemic series (Table 5), probably indicating again that these patients are on the PIMS-TC spectrum.

Surprisingly, there is an absence of reported cases of Kawasaki-like MIS associated with SARS-CoV-2 infection in Asian countries where the COVID-19 pandemic started, and where the incidence of KD was the highest. It has been hypothesized that a mutation from a European strain of SARS-CoV-2 drives a stronger cytokine storm. Serology was more likely to be positive than PCR in our series, supporting the postinfectious hypothesis, because the disease appears to occur 2–4 weeks after acute SARS-CoV-2 infection or exposure. This has also been observed in the patients with PIMS-TC and those with heart failure in this context (23, 25–27). The coronavirus family might represent one of the triggers of KD as previously hypothesized (32, 33), and the SARS-CoV-2 spike may act as a superantigen driving a cytokine storm that leads to hyperinflammation (14, 34). A positive microbiological finding other than SARS-CoV-2 was found in around 20–25% of the PreCoV and CoV– patients. Some of the CoV– negative patients could have had a different trigger despite the lockdown, but some may have been CoV false negatives explaining why this group has overlapping clinical and laboratory features. There were no meaningful differences in blood results between PreCoV and CoV– patients. Nevertheless, when comparing PreCoV to CoV+ patients, we found significantly lower leucocyte and platelet count and higher NT-proBNP, ferritin, CRP, and PCT in CoV+ patients as found in other cohorts (13, 23). These findings are closer to those exhibited by the PIMS-TC/MIS-C and KDSS (35) patients than to classical KD patients. The NT-proBNP levels we found were more in line with the Paris Kawa-COVID-19 cohort (median 4,319 pg/mL) (15) in contrast with Belhadjer et al. that reported 10 times higher levels (mean 41,484 pg/mL) in PIMS-TC patients (26).

Echocardiographic examinations were abnormal in 50% of CoV+ patients similar to other KD pandemic series (Table 5) and higher than classical KD cases; this is probably because many CoV+ patients presented with left ventricular dysfunction. CAL, defined as Z-score > 2, were observed in a third of our patients presenting during the pandemic, but <10% had coronary aneurysms, and this was less likely in the CoV+ group. In PIMS-TC patients, 7–12% present aneurysms (25). Interestingly, there are at least three studies that report no coronary aneurysms in Kawa-COVID-19 patients (15) and PIMS-TC patients (23, 26); in absolute number, we only had one patient in the CoV+ group with a coronary aneurysm. Our impression is that the cases associated with SARS-CoV-2, and although they have more cardiac involvement, it is more likely to be left ventricular dysfunction than cardiac aneurysms, but this needs to be studied further. It is known that older children with KD and intense inflammation are more likely to present with myocarditis (36). Children with PIMS-TC have been described to have mild to moderate heart dysfunction indicating acute myocardial injury and recovering normal cardiac function within a week, probably because there is inflammation and myocardial oedema but without myocardial necrosis (31, 37). Left ventricular dysfunction was the only echocardiographic finding that was significantly higher in CoV+, and all cases were transient. Abnormal repolarization was relatively frequent in CoV+ patients in our study; this is probably linked to the higher proportion of patients with left ventricular dysfunction. A range of ECG abnormalities (14–60%) have been reported in PIMS-TC patients (5, 25).

We found that while 100% of CoV– patients received IVIG, this drops to 80% in the CoV+ patients. We think this is because some may have been treated as PIMS-TC/MIS-C where some clinicians gave steroids directly or they recovered with supportive care alone. Although there are no studies yet on the best treatment for this new entity, most groups are giving first-line treatment with IVIG followed by steroids in some patients ranging from 33 to 64%, and biological agents in 8–14% (5, 23, 25, 26). A second IVIG dose was given in a similar proportion of patients from all groups, and corticosteroids were given more frequently in CoV+ patients, probably due to higher inflammation markers and suspicion of PIMS-TC.

A significantly higher number of patients were admitted to PICU from the CoV+ group. The reason for PICU admission was due to cardiac dysfunction or shock resembling again PIMS-TC rather than classical KD (5, 23, 25). The mean length of the PICU stay was generally under a week (4–5 days) (23, 25, 27). Only one patient from PreCoV had a persistent aneurysm, and one in the CoV- is still under follow-up. No deaths were reported.

Our study has some potential limitations. There is a potential recruitment bias that may have contributed to the high number of patients with Kawasaki-like multisystem inflammatory syndrome, as pediatricians have been more aware due to the alerts. Antibody tests against SARS-CoV-2 were performed by different techniques depending on the Hospital; therefore, sensitivity and specificity have a broad range, driving to a potential underdiagnosis. We could not calculate an overall incidence of KD because our network did not achieve total national coverage. Nevertheless, we think that the number of patients collected allows us to draw conclusions, and the prospective character of our network KAWA-RACE before and during the pandemic places us in a privileged setting to have an overview of what has happened in Spain.

In summary, we describe findings in the CoV+ group and remark clinical and laboratory differences to classify them as PIMS-TC/MIS-C and not classical KD (higher median age, non-Caucasian predisposition, more likely incomplete presentation, more myocardial dysfunction, less aneurysm development, more PICU admission, and higher inflammatory markers). Whether this is a novel entity or the same disease on different ends of the spectrum is yet to be elucidated. However, SARS-CoV-2 seems to be a potent trigger that in some patients leads to an aberrant immune response, especially in older children, and may be due to previous infections.

## Data Availability Statement

The raw data supporting the conclusions of this article will be made available by the authors, under reasonable request.

## Ethics Statement

The studies involving human participants were reviewed and approved by Instituto de Investigacion Hospital 12 de Octubre approved this study (CEIC 15/316). Written informed consent to participate in this study was provided by the participants' legal guardian/next of kin.

## Author Contributions

EF-C, CG, ABa, JS-M, BM, EV, MC, MN, MO, GG, MB, ABe, BR, MR-G, EN, JAr, and DM contributed to the acquisition of data. EF-C, CG, SD-R, ABa, JS-M, JAn, and CC contributed to the analysis and interpretation of the data and drafted the manuscript. EF-C, CG, ABa, JS-M, JAn, and CC contributed to the conception and design of the manuscript. BM, EV, MC, MN, MO, GG, MB, ABe, BR, MR-G, EN, JAr, and DM critically revised the manuscript. All authors gave final approval and agreed to be accountable for all aspects of work ensuring integrity and accuracy.

## Conflict of Interest

The authors declare that the research was conducted in the absence of any commercial or financial relationships that could be construed as a potential conflict of interest.

## KAWA-RACE Study Group Collaborative Authors

**The KAWA-RACE study group is composed by:**

**Study coordinator:** Elisa Fernández-Cooke (Hospital Doce de Octubre, Spain).

**Scientific committee:** Elisa Fernandez-Cooke (Hospital 12 de Octubre, Madrid), Cristina Calvo (Hospital Universitario La Paz, Madrid), Ana Barrios Tascón (Hospital Universitario Infanta Sofia, Madrid), Judith Sánchez-Manubens (Hospital Sant Joan de Deu, Barcelona), Jordi Antón (Hospital Sant Joan de Deu, Barcelona), Javier Aracil Santos (Hospital Universitario La Paz, Madrid), Esmeralda Nuñez Cuadros (Hospital Regional Universitario de Malaga), Maria Luisa Navarro Gómez (Hospital Universitario Gregorio Marañon, Madrid), David Moreno Pérez (Hospital Regional Universitario de Málaga), Carlos Grasa Lozano (Hospital Universitario La Paz, Madrid).

**Collaborating members:**

María Martín Cantero Pérez (Hospital Regional Universitario de Málaga), Esmeralda Nuñez Cuadros Pérez (Hospital Regional Universitario de Málaga), Begoña Carazo Gallego Pérez (Hospital Regional Universitario de Málaga), Fernando Sánchez García (Hospital La Inmaculada, Almería), Marisol Camacho Lovillo (Hospital Virgen del Rocío, Sevilla), Renata Marqués (Hospital Virgen del Rocío, Sevilla), Olaf Neth (Hospital Virgen del Rocío, Sevilla), Laura, Fernández Silveira (Hospital Virgen del Rocío, Sevilla), Miguel Sánchez Forte (Hospital Torrecardenas, Almería), Ángeles Ortega Montes (Hospital Torrecardenas, Almería), Leticia Isabel Martínez Campos (Hospital Torrecardenas, Almería), Beatriz Bravo Mancheño (Hospital Universitario Virgen de las Nieves, Granada), Margarita Camacho (Hospital Universitario Virgen de las Nieves, Granada), Antonio F Medina Claros (Hospital de la Axarquía, Murcia), Carlos Salido (Hospital de Jerez), María Torres Rico (Hospital Juan Ramón Jiménez de Huelva), Beatriz Ruiz Saez (Hospital Reina Sofia de Córdoba), Elena Fernadez de la Puebla Lechuga (Hospital Reina Sofia de Córdoba), Ma José Lirola Cruz (Instituto Hispalense de pediatría, Sevilla), Kety Maya Carrasco (Instituto Hispalense de pediatría, Sevilla), Moisés Rodríguez González (Hospital Universitario Puerta Del Mar de Cádiz), Enrique Blanca Jover (Complejo Hospitalario Granada), José Uberos Fernández (Complejo Hospitalario Granada), María Mercedes Ibáñez Alcalde (Hospital de Poniente, Almería), Miguel Lafuente Hidalgo (Hospital Universitario Miguel Servet, Zaragoza), Lorenzo Jiménez Montañés (Hospital Universitario Miguel Servet, Zaragoza), Matilde Bustillo Alonso (Hospital Universitario Miguel Servet, Zaragoza), Ariadna Ayerza Casas (Hospital Clínico Universitario Lozano Blesa, Zaragoza), Bárbara Montes Zapico (Hospital San Agustín, Avilés), Carlos Pérez Méndez (Hospital Universitario de Cabueñes, Gijón), Javier Fernández Aracama (Hospital Universitario Basurto, Bilbao), Lucía Rodríguez (Hospital Central de Asturias, Oviedo), María Aleida Ibáñez Fernández (Hospital Central de Asturias, Oviedo), Sandra Navarro Campo (Hospital Central de Asturias, Oviedo), Silvia Escribá Bori (Hospital Universitario Son Espases, Palma de Mallorca), María Concepción Mir Perelló (Hospital Universitario Son Espases, Palma de Mallorca), Ma Ángeles de la Fuente Sánchez (Hospital Universitario Son Espases, Palma de Mallorca), Patricia Aparicio García (Hospital Son Llátzer, Palma de Mallorca), Carlos Briales (Hospital Son Llátzer, Palma de Mallorca), Joaquín Castilla Crespí (Hospital Son Llátzer, Palma de Mallorca), María Elena Colino Gil (Hospital Materno-Infantil de Las Palmas de Gran Canaria), Nerea Delgado Cabrera (Hospital Materno-Infantil de Las Palmas de Gran Canaria), Ana Bello Naranjo (Hospital Materno-Infantil de Las Palmas de Gran Canaria), Jesús Poch Páez (Hospital Materno-Infantil de Las Palmas de Gran Canaria), Moneyba García Yáñez (Hospital Materno-Infantil de Las Palmas de Gran Canaria), Montse González García (Hospital Universitario Nuestra señora de Candelaria de Santa Cruz de Tenerife), Maite Viadero (Hospital Universitario Marqués de Valdecilla, Santander), Beatriz Jiménez Montero (Hospital Universitario Marqués de Valdecilla, Santander), Olga Domínguez García (Hospital Virgen de la Salud de Toledo), Begoña Losada Pinedo (Hospital Virgen de la Salud de Toledo), Gema Iñigo Martín (Hospital Virgen de la Salud de Toledo), Lucía María Escribano Gómez (Hospital General de Albacete), Antonio Cepillo (Hospital General de Albacete), Miguel Lillo Lillo (Hospital General de Albacete), María Isabel Buedo (Hospital General de Albacete), Laura del Rey (Hospital General de Albacete), Elena Urbaneja Rodríguez (Hospital Clínico Universitario de Valladolid), Sara Rellán Rodríguez (Hospital Clínico Universitario de Valladolid), Teresa Cantero (Hospital de Palencia), Beatriz Plata Izquierdo (Complejo Asistencial Universitario de Salamanca), Luisa García-Cuenllas Álvarez (Complejo Asistencial Universitario de Salamanca), Ignacio Oulego Erroz (Hospital de León), Elena Pérez Santaolalla (Hospital Universitario de Burgos), Carlos Alcalde Martín (Hospital Universitario Río Hortega de Valladolid), Fernando Centeno Malfaz (Hospital Universitario Río Hortega de Valladolid), Elena Pérez Gutiérrez (Hospital Universitario Río Hortega de Valladolid), Ma Soledad Jiménez Casso (Hospital General de Segovia), Fredy Prada (Hospital Sant Joan de Déu, Barcelona), Rosa Bou (Hospital Sant Joan de Déu, Barcelona), Estibaliz Iglesias (Hospital Sant Joan de Déu, Barcelona), Joan Calzada (Hospital Sant Joan de Déu, Barcelona), Olga Calavia Garsaball (Hospital Joan XXIII de Tarragona), Marc Tobeña Rue (Hospital Vall d'Hebron, Barcelona), Gemma Giralt García (Hospital Vall d'Hebron, Barcelona), Zulema Lobato (Xarxa Assistencial i Universitaria de Manresa), Neus Rius Gordillo (Hospital Sant Joan de Reus), Montserrat Pascual Torres (Hospital Sant Joan de Reus), María Méndez Hernández (Hospital Germans Trias i Pujol de Badalona), Lourdes García (Hospital de Mataró), Sergio Flores Villar (Hospital Universitari Mútua de Terrassa), Silvia Yevenes Ruiz (Hospital Universitari Mútua de Terrassa), Laura Minguell Domingo (Hospital Arnau de Vilanova, Lleida), Anna Ballester (Hospital de Calella), Ana Miralles (Hospital de Palamós), Berta Pujol Soler (Hospital General de Granollers), Anton Foguet Vidal (F. Hospital d'Olot Comarcal de la Garrotxa), Pere Sala Castellví (Hospital de Barcelona), Angelita Serrano Aguiar (Hospital Comarcal Mòra d'Ebre), José Manuel Siurana Rodríguez (Hospital HM Nens. HM Hospitales), Anna Sangorrin Iranzo (Hospital HM Nens. HM Hospitales), Roser Álvarez Pérez (Hospital de la Santa Creu i Sant Pau de Barcelona), Paula Ribes Cajas (Hospital General de Catalunya), Pere Genaró i Jornet (Hospital Verge de la Cinta, Tortosa), Ana Grande Tejada (Complejo Hospitalario Universitario Infanta Cristina, Badajoz), Cristina Zarallo (Complejo Hospitalario Universitario Infanta Cristina, Badajoz), Federico Martinón-Torres (Hospital Clínico Universitario de Santiago de Compostela), Irene Rivero Calle (Hospital Clínico Universitario de Santiago de Compostela), Antonio Justicia Grande, (Hospital Clínico Universitario de Santiago de Compostela), María López Sousa (Hospital Clínico Universitario de Santiago de Compostela), Alejandro Souto Vilas (Hospital Clínico Universitario de Santiago de Compostela), Bernardo López Abel (Hospital Clínico Universitario de Santiago de Compostela), Elisa de Miguel Esteban (Hospital San Pedro, Logroño), Bibiana Riaño Méndez (Hospital San Pedro, Logroño), Daniel Blázquez (Hospital Universitario Doce de Octubre, Madrid), Pablo Rojo Conejo (Hospital Universitario Doce de Octubre, Madrid), Belén Toral (Hospital Universitario Doce de Octubre, Madrid), Leticia Albert De la Torre (Hospital Universitario Doce de Octubre, Madrid), Jaime de Inocencio (Hospital Universitario Doce de Octubre, Madrid), Mar Santos (Hospital Universitario Gregorio Marañon, Madrid), Rafael Díaz-Delgado de la Peña (Hospital Severo Ochoa, Leganés), Paz Collado Ramos (Hospital Severo Ochoa, Leganés), Alfredo Tagarro (Hospital Universitario Infanta Sofia, Madrid), Teresa Raga (Hospital Universitario Infanta Sofia, Madrid), Libertad Latorre (Hospital Universitario Infanta Sofia, Madrid), Sara Guillén (Hospital de Getafe), Ignacio Callejas Caballero (Hospital de Getafe), Luis Manuel Prieto Tato (Hospital 12 de Octubre), María Fernanda Guzmán Monagas (HM Hospitales Madrid), Isabel Jiménez López (HM Hospitales Madrid), Sandra Villagrá (HM Hospitales Madrid), Viviana Arreo (HM Hospitales Madrid), Roi Piñeiro Pérez (Hospital General de Vilalba), María de la Parte (Hospital General de Villalba), Amalia Tamariz-Martes (Hospital Infantil Universitario Niño Jesús, Madrid), Marta Llorente Romano (Hospital del Sureste, Madrid), Maria Belén Hernández Rupérez (Hospital del Sureste, Madrid), Henar Rojo Sombrero (Hospital Universitario Príncipe de Asturias, Alcalá de Henares), Estefanía García Cerro (Hospital Universitario Príncipe de Asturias, Alcalá de Henares), Irene Maté Cano (Hospital del Henares, Coslada), Marta Villares Alonso (Hospital de Móstoles), Marta Pilar Osuna Marco (Hospital de Móstoles), Julia Jensen Veron (Hospital Infanta Cristina, Madrid), Cristina Zarallo Reales (Hospital Infanta Cristina, Madrid), María Dolores Rodríguez Mesa (Hospital Infanta Cristina, Madrid), Santiago Rueda Esteban (Hospital Clínico San Carlos, Madrid), José Tomás Ramos Amador (Hospital Clínico San Carlos, Madrid), Cristina González Menchén (Hospital Clínico San Carlos, Madrid), Ana Belén Jiménez Jiménez (Hospital Universitario Fundación Jiménez Díaz, Madrid), Pilar Galán (Hospital Universitario de Fuenlabrada), Dolores Pérez Campos (Hospital Universitario de Fuenlabrada), Ma Mercedes Bueno (Fundación Hospital de Alcorcón), David Crespo Marcos (Fundación Hospital de Alcorcón), Enrique Otheo de Tejada Barásoain (Hospital Ramón y Cajal, Madrid), Walter Alberto Sifuentes Giraldo (Hospital Ramón y Cajal, Madrid), María Luz Gámir Gámir (Hospital Ramón y Cajal, Madrid), María José Cilleruelo Ortega (Hospital Puerta de Hierro, Majadahonda), Agustín López López (Hospital Puerta de Hierro, Majadahonda), Cristina Sánchez Vaquerizo (Hospital Puerta de Hierro, Majadahonda), Ana Isabel Usano Carrasco (Hospital Puerta de Hierro, Majadahonda), Ester Moreno Gómez (Hospital San Rafael, Madrid), Olga Carvajal del Castillo (Hospital Rey Juan Carlos, Móstoles), Beatriz Del Pozo Menéndez Hospital de Torrejón), Katie Badillo Navarro (Hospital de Torrejón), Fernando Baquero (Hospital Universitario La Paz, Madrid), Lucía Deiros Bronte (Hospital Universitario La Paz, Madrid), Pablo Fernández Fraga (Hospital Universitario La Paz, Madrid), Nieves Domínguez (Hospital Universitario Infanta Elena, Madrid), Beatriz Mercader Rodríguez (Hospital Clínico Universitario Virgen de la Arrixaca, Murcia), Francisco Castro García (Hospital Clínico Universitario Virgen de la Arrixaca, Murcia), Águeda Herrera Chamorro (Hospital Clínico Universitario Virgen de la Arrixaca, Murcia), Paula Alcañiz Rodríguez (Hospital Clínico Universitario Virgen de la Arrixaca, Murcia), Moisés Sorlí García (Hospital Clínico Universitario Virgen de la Arrixaca, Murcia), María Concepción Rex Nicolás (Hospital Universitario Santa Lucía, Cartagena), Elena Vera Romero (Hospital Universitario Santa Lucía, Cartagena), Patricia Martínez Olorón (Complejo Hospitalario de Navarra), Beatriz Rocandio Cilveti (Hospital de Donostia), Amaia Berridi (Hospital de Donostia), Laura Santos-Díez Vázquez (Hospital de Cruces, Bilbao), Olaia Fernández (Hospital Universitario Basurto, Bilbao), Inmaculada Calvo (Hospital Universitario y Politécnico La Fe, Valencia), Belén Fernández Tudela (Hospital Universitario y Politécnico La Fe, Valencia), Manuel Oltra Benavent (Hospital Universitario y Politécnico La Fe, Valencia), Marta Dapena Archilés (Hospital General de Castellón), Franciasco Sanchez Ferrer (Hospital Universitario de San Juan de Alicante), César Gavilán (Hospital Universitario de San Juan de Alicante), Ignacio Izquierdo Fos (Hospital Universitario de Elche), María Isabel Serrano Robles (Hospital Universitario de Elche), Yolanda Herranz Sánchez (Hospital Marina Baixa de Alicante), Enrique Villalobos Pinto (Hospital Infantil Universitario Niño Jesús, Madrid), Daniel Clemente Garulo (Hospital Infantil Universitario Niño Jesús, Madrid), Stella Pie (Hospital Universitari Doctor Josep Trueta, Girona), Manuel Marrero Calvo (Hospital Nuestra Señora de Sonsoles, Ávila), José María Olmos García (Hospital Mare de Déu dels Lliris, de Alcoy, Alicante).
